# Sentinel Lymph Node Mapping in Breast Cancer: Initial Experience of a Multidisciplinary Team

**DOI:** 10.7759/cureus.25983

**Published:** 2022-06-16

**Authors:** Tahira Yasmin, Muhammad Numair Younis, Misbah Masood, Huma Majeed Khan, Zahid Asgher, Abubaker Shahid

**Affiliations:** 1 Nuclear Medicine and PET Imaging, Institute of Nuclear Medicine & Oncology (INMOL) Cancer Hospital, Lahore, PAK; 2 Clinical Oncology, Institute of Nuclear Medicine & Oncology (INMOL) Cancer Hospital, Lahore, PAK; 3 Breast Surgery, Ittefaq Hospital (Trust), Lahore, PAK; 4 Pathology, Lahore Medical and Dental College, Lahore, PAK; 5 Oncology, Institute of Nuclear Medicine & Oncology (INMOL) Cancer Hospital, Lahore, PAK

**Keywords:** breast cancer, single-photon emission computerized tomography, spect-ct, lymphoscintigraphy, sentinel lymph node, breast carcinoma, radionuclide imaging

## Abstract

Introduction: Breast cancer is one of the leading causes of cancer-related deaths in women; it is the most frequently diagnosed cancer in women in the United States with a lifetime risk of dying of about 3.4%. Regional lymph node involvement is quite early in breast carcinoma and axillary lymph node metastasis is an important predictor of recurrence and survival, particularly in invasive ductal histology of breast carcinoma. Localization of sentinel lymph node/nodes followed by frozen section and histopathological evaluation helps to prevent unnecessary axillary nodal dissection and, hence, reduces associated post-surgical morbidity. Sentinel nodes are the first ones to receive lymph-borne metastatic cells and, hence, lymphoscintigraphy followed by biopsy is quite reliable to detect nodal metastasis, particularly at an early stage (I, II) of breast cancer.

Methods: Here we will share our experience of introducing procedure, personnel training, and workflow of sentinel lymph node lymphoscintigraphy in breast cancer patients at our cancer institute to help other centers establish programs for this study.

Results: Initially, 10 procedures were performed, all of which were successful in the localization of sentinel nodes and played a substantial part in the surgical planning of breast cancer. Planar lymphoscintigraphy and single-photon emission computerized tomography (SPECT)-CT images of our first patient revealed radiotracer avidity in the lymph node in the ipsilateral axilla, which was later on diagnosed as metastatic resulting in axillary nodal clearance.

Conclusions: In multidisciplinary/closely-placed surgical, pathological, and hybrid imaging facility settings, lymphoscintigraphy provides a quick, accurate, and better way of nodal localization leading to correct surgical decision-making. In addition to planar imaging, SPECT-CT acquisition significantly improves the specificity of the lymphoscintigraphy procedure, which is beneficial for patients to avoid false-positive results, thus saving breast cancer patients from potential adverse effects of surgery.

## Introduction

Breast cancer is one of the leading causes of cancer-related deaths in women; it is the most frequently diagnosed cancer in women in the United States with a lifetime risk of dying of about 3.4% [1]. Over the years, breast cancer incidence has significantly increased among Asian women as well. In the year 2012, more than 600,000 new breast cancer cases were reported in Asia accounting for 39% of all breast cancers diagnosed worldwide [2] and in 2018, approximately 839,000 women were diagnosed with breast cancer and over 286,000 died of the disease in the region [3].

Regional lymph node involvement is quite early in carcinoma breast and axillary lymph node metastasis is an important predictor of recurrence and survival, particularly in invasive ductal histology of breast carcinoma. Early diagnosis plays a vital role in a better prognosis and improved management; therefore, accurate staging and identification of metastatic lymph nodes are critical. Due to uncertain stage of disease, axillary lymph node dissection was a routine clinical practice with the aim of reducing recurrence risk and increasing the rate of survival; however, with the development of advanced radiologic tools, correct staging is now possible. The radionuclide imaging technique of sentinel lymph basin identification and localization is quite well known and is being utilized in almost all parts of the world, and successful localization of sentinel lymph node (SLN) is possible in almost 95% of breast cancer patients [4]. Localization of SLN/SLNs followed by frozen section and histopathological evaluation helps to prevent unnecessary axillary nodal dissection and, hence, reduces associated post-surgical morbidity. Another benefit of axillary lymph node sampling guided by SLN biopsy over axillary lymph node dissection is the fact that fewer nodes are resected in the former procedure compared with the latter, allowing for more detailed scrutiny of the resected nodes for malignant cells. SLNs are the first ones to receive lymph-borne metastatic cells and lymphoscintigraphy followed by biopsy is quite reliable to detect nodal metastasis, particularly at an early stage (I, II) of breast cancer.

Initially, the injection of radio-colloid around the primary breast lesion, its progression along the lymphatic channels, and phagocytosis by macrophages in sentinel nodes is the basis of imaging with the planar gamma camera; however, utilization of a hand-held gamma probe sensitive to the emissions of the radio-colloid is quite common and helpful in exact localization of SLN followed by frozen section and histopathologic diagnosis. Lymphoscintigraphic imaging prior to surgery has certain benefits over hand-held gamma probe as it improves accuracy and reduces morbidity; it may also act as a check on the use of appropriate radiotracer and radiopharmaceutical injection failure. Depending upon the natural variability in breast lymphatic of patients, lymphoscintigraphy mapping may be done to identify SLNs in axillary and intra-thoracic nodal sites. Early planar gamma camera images mostly lead to the identification of the first draining lymph nodes as a sentinel by visualization of their lymphatic ducts while secondary lymph nodes can be differentiated from primary as the former appear on delayed images. The sensitivity and specificity of imaging techniques are further enhanced by single-photon emission computed tomography (SPECT)-CT acquisition of single or multiple regions. SPECT-CT imaging gives detailed information and exact anatomic localization of already visualized sentinel nodes on planar images; it also detects lymph nodes in the vicinity of primary breast lesions and in areas of complex anatomy like the head and neck region [5].

Here, we will share our experience of introducing SLN lymphoscintigraphy in breast cancer patients at our cancer institute. Although ours is a well-established public sector cancer facility with state-of-the-art nuclear medicine (dual-head gamma cameras with SPECT-CT, cyclotron, and positron emission tomography (PET)-CT scanners), radiology (Aplio USG machine, 64 slices CT, 6T MRI; Canon Medical Systems Corporation, Otawara, Tochigi, Japan), radiotherapy (three linear accelerators (LINACs), intensity-modulated radiation therapy (IMRT)), chemotherapy bay, radioimmunological assay (RIA) laboratory, and clinical lab with advanced equipment, yet radionuclide SLN imaging took us a long time to begin with, probably because of less frequent referrals. SLN lymphoscintigraphy in breast cancer patients is already in practice in one of the public sector hospitals in Islamabad and two private-sector cancer hospitals, one in Karachi and the other in our province; however, we are the first ones among public sector hospitals in this region. There was an increasing demand by classified breast surgeons to perform SLN lymphoscintigraphy for breast cancer patients.

There are always financial constraints in the case of public sector organizations; however, our institute is capable of smoothly running finances of smaller-scale activities. Detailed departmental meetings of senior consultants, technologists, and the head of the nuclear medicine department were held to decide and nominate the existing facilities and equipment (history room, injection area, gamma camera) to be utilized, equipment (nano-colloid kits, emergency tray with all emergency medicine and tools, surgical gowns, surgical gloves, disposable insulin syringes, screens, disinfectant, gauze packs) to be purchased, type of radiotracer and its availability (globally utilized 99mTc, already available at the institute), a dose of radiotracer and nano-colloid particles, staffing (doctors, technologists, ward attendants), and specified days for the procedure without disturbing routine imaging procedures being performed on daily/weekly basis. 

## Materials and methods

This study presents a short case series that was ethically approved by the Institute of Nuclear Medicine & Oncology (INMOL) Institutional Review Board, Lahore, Pakistan (approval number 05-11/21). The study was carried out at the Institute of Nuclear Medicine & Oncology (INMOL) hospital, Lahore, Pakistan.

Devising the procedure

The study procedure was devised according to the European Association of Nuclear Medicine (EANM) and Society of Nuclear Medicine and Molecular Imaging (SNMMI) practice guidelines for lymphoscintigraphy and SLN localization in breast cancer 2013 [6], which are considered standard operating procedure (SOP) guidelines; these are usually common practice in hospitals already performing this study. It’s important to initially make a feasibility report by discussing and analyzing structural and operational costs, all required equipment, method of purchase, and associated logistics issues. It is recommended to arrange procedural steps in such a way that outcome presents all the information required by referring surgeons. The first step was to train the related staff at the already practicing center; as the procedure is not much complicated, only two to three weeks of training for doctors and technologists was sufficient. SOPs included the patient’s visit to the hospital a day or two before the procedure and clinical assessment (history, examination of site of lesion and axillae), evaluation of previous investigations or imaging procedures available, instructions (following timeline, clean site of lesion, making the patient understand the injection technique with possible associated pain, reassurance), and procedure cost. The essentials of SLN lymphoscintigraphy procedure are given in Table 1.

**Table 1:** Essentials of SLN lymphoscintigraphy procedure SLN: sentinel lymph node; NM: nuclear medicine; HSA: human serum albumin; Tc: technitium; Mo: molybdenum Xeleris: Xeleris™ (GE Healthcare, Chicago, Illinois, United States)

Serial No	Staff	Consumables	Radio-pharmaceutical	Facilities	Medicines
1	Receptionist	Nano-colloid kits	^99m^Technitium	Clinical assessment room	Adrenaline
2	Doctor	Emergency medicine and tools	HSA-nanocolloid	Source of radiotracer (Mo-Tc generator)	Disinfectant solution
3	Radio-pharmacist	Surgical gowns & gloves	-	Elution facility (Fume-hood)	Local anesthetics
4	Radio-chemist	Disposable insulin syringes	-	Dose dispensing area	-
5	NM Technologist	Screens	-	Dual head gamma camera	-
6	Attendant/ ward boy	Gauze packs	-	Image interpretation soft-wares (Xeleris)	-

One-day (SLN lymphoscintigraphy procedure on the day of surgery/frozen section) or two-day (SNL lymphoscintigraphy one day before surgery/frozen section) protocols are being exercised worldwide. Depending upon the patients' availability and planned surgical procedure, same-day SLN imaging is quite feasible. In our study, on the day of surgery (planned in the evening), the patients were advised to visit for SLN imaging early in the morning. After recording history details and local clinical examination, patients was ready to be injected. Injections were administered on the imaging table followed by dynamic (30min) and static imaging (after every 15 min) till SLN visualization. SPECT-CT acquisition of the chest and neck region was also performed. After the study reporting, they were discharged with as low as reasonably achievable (ALARA ) instructions regarding radioactive exposure. 

Staffing

The leader in this procedure is nuclear medicine consultant who has expertise in understanding and performing radionuclide imaging procedures and is trained in SLN lymphoscintigraphy technique. Usually, the doctor decides the dose and is responsible for aseptic conditions and accurate injection technique, and the same was followed in our settings. Image interpretation and reporting were done by a team of two to three nuclear medicines consultants.

A technical member of the team was deputed for the purchase of required equipment (nano-colloid kits), kit maintenance, and record-keeping. He was also involved in image acquisition and data processing. Another staff member (nurse/assistant technologist) was assigned to prepare the patient for the injection.

Patient preparations

Written informed consent of the patients was taken prior to enrollment in the study. No specific patient preparation was considered necessary before arrival at the hospital; however, the patients were advised to clean the probable site of injection and wear loose and comfortable clothing. Prior to dose administration, clothing and all jewelry above the waist were removed and patients were allowed to wear front open gowns. Images and reports of previously performed breast and axillae ultrasound, mammograms, CT, or MRI were available to be assessed by nuclear medicine doctor.

Dose preparation

Human serum albumen (HSA)-based kit, Nanotop® HSA nano-colloid kit (ROTOP Pharmaka GmbH, Dresden, Germany) was used that contained particle size ranging between 50 to 80 nm. Technetium-99m (99mTc) was obtained in the required amount as pertechnetate (TcO4) from freshly eluted molybdenum-99 (99Mo)-99mTc generator and added to a nano-colloid kit. Radiotracer activity and solution volume were kept controlled in such a way that each syringe contained 0.2 ml solution and the total dose administered to the patient was 30 MBq for the same-day procedure and up to 150 MBq for the two-day protocol. Quality control procedure was performed by radiochemistry experts a few minutes before dose administration. The radiochemical purity of the radiopharmaceutical was checked by paper chromatography (instant thin-layer chromatography (ITLC)) by using silica gel (SG-60) in acetone solvent.

Injection techniques

**Figure 1:**
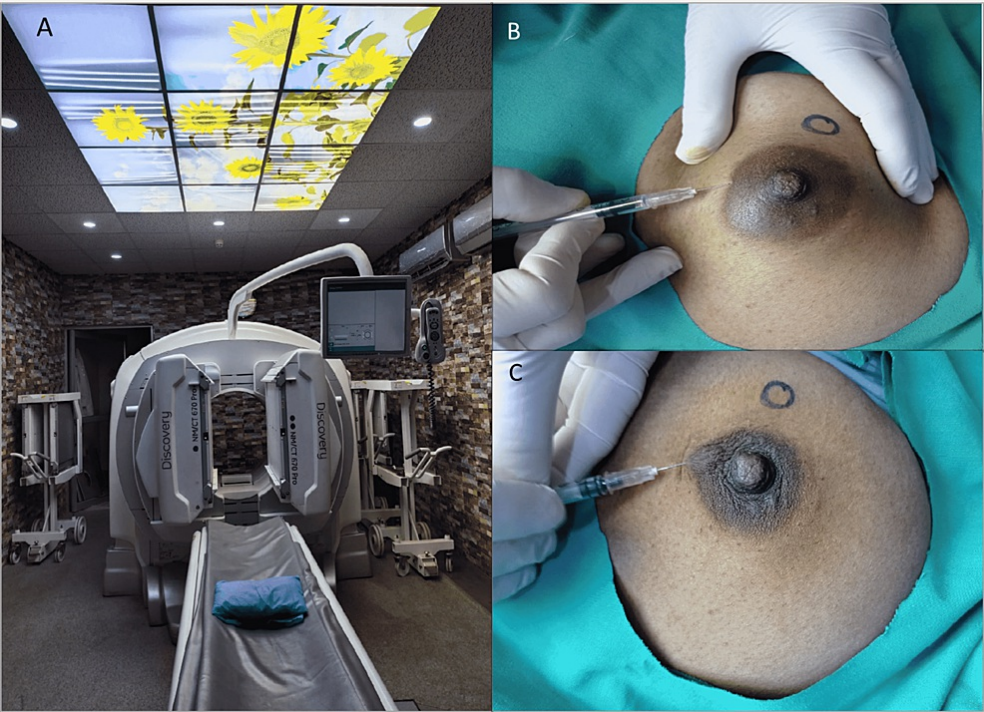
A: SPECT-CT gamma camera; B,C: Peri-areolar injection technique SPECT: single-photon emission computerized tomography

There are different methods of nano-colloid injection in the vicinity of the primary lesions. As shown in Figure 1 (panels B, C), the peri-areolar technique is the most widely used one; it involves two to four syringes (preferably small size for example insulin syringes) each containing 0.05-0.5 ml of dose solution. Volumes are kept low as larger volumes can interfere with the normal lymphatic flow, injected in the peri-areolar region, intradermal or subcutaneous [6]. In the case of peri-tumoral injection, the dose is injected in breast tissue surrounding the base of the primary lesion, it is a deeper approach and is mostly utilized to visualize internal mammary lymph nodes. However, both the techniques have strengths and drawbacks and it does not appear to make sufficient difference in management, whether superficial or deep. Because of easy access, the more superficial and rapid localization of axillary lymph nodes peri-areolar technique turned out to be the technique of our choice. Three syringes were used to inject nano-colloid intra-dermally in the peri-areolar region followed by a gentle massage at injection sites to augment rapid and smooth flow through lymphatic channels. 

Image acquisition protocol

Although our referee had the facility of peri-operative hand-held gamma probe utilization, we decided to image the lymphatic on a dual-head gamma camera (Discovery™ 670, GE Healthcare, Chicago, Illinois, United States) (Figure 1A)with SPECT and 64-slice CT (Figure 1A) on account of the above-mentioned benefits. Daily routine quality control check was performed prior to imaging. Acquisition protocol included the use of low-energy, high-resolution (LEHR) collimator and energy window 20% centered at 140 KeV photopeak, immediately after radiopharmaceutical injection dynamic imaging (1 minute/frame) was performed for the first 30 minutes followed by serial static images every 15 minutes till visualization of probable SLN with camera heads at an anterior and lateral position to the affected breast. Study acquisition was stopped at 120 min after injection in case no nodal radiotracer accumulation was detected. For anatomical localization, planar imaging was followed by SPECT-CT acquisition of the chest, including axillae and lower neck. SPECT images were acquired by step and shoot method with 128 X 128 matrix prior to low dose CT chest. During the acquisition process (planar and SPECT) patient's position was the same (supine). We did not acquire further images with additional patient positions and oblique (left anterior oblique (LAO), 450) views owing to already planned SPECT-CT imaging. 

Image archiving and interpretation

Proper image archiving systems and building databases are crucial to avoid data loss, which may be helpful for patients as well as researchers to be utilized for further changes and improvements. We have three sets of image storing systems/software, including GE software for raw data storage at the console, processed images save screens at the processing PC (Xeleris™ 4 D, GE healthcare), and inter-departmental institutional picture archiving and communication system (PACS) database. Lymphoscintigraphic images were stored at all three systems. Planar images do not require any significant processing; however, injection sites, primary and secondary sentinel nodes, and lymphatic channels if visualized are marked on these images. Processing, required fusion, and the above-mentioned marking of SPECT and CT images were done at the Xeleris system (GE Healthcare).

Image interpretation and processing were performed jointly by a team of three nuclear medicine doctors with more than five years of experience as nuclear medicine specialists, one senior consultant with expertise in SPECT-CT image reading, and two juniors.

Study reporting

Our reporting format is based on the one described by EANM/SNMMI guidelines 2013 [6]. It outlines patient particulars with contact information, details of the radiopharmaceutical, dose, activity volumes, injection site, and imaging protocols as described above. Brief clinical history of the patient was also mentioned. The body of the report clearly demonstrated the site and time of localization of SLN/SLNs and additional findings including visualization of lymphatic channels were also mentioned.

Communication with referring surgeon

The referring surgeon was informed about the completion of the procedure immediately after image processing and visualization of the SLN and all related planar and SPECT-CT images were shared through personal contacts beforehand so that early preparations for the frozen section are made. A formal written report was ready 30 minutes after study completion and it was made available for patients for collection at a suitable time. Strong communication between the nuclear medicine department and specified surgical facility with an active communication medium is required to make lymphoscintigraphy mapping and conservative axillary nodal clearance successful.

## Results

**Figure 2:**
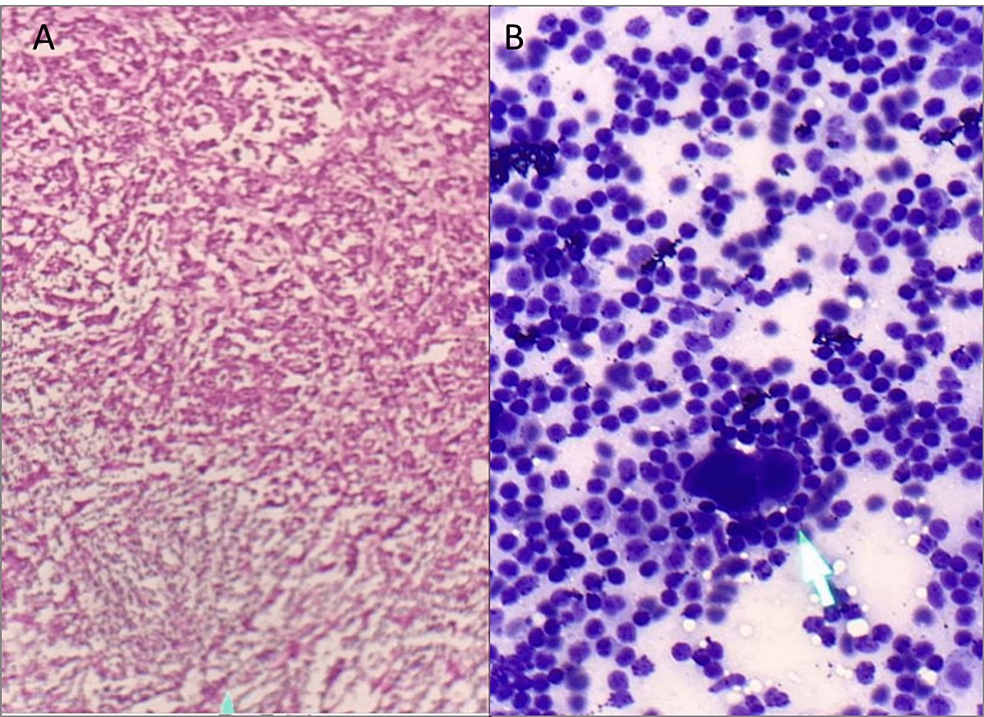
A: Frozen section of lymph node reveals metastatic involvement; B: Touch preparation of lymph node shows metastatic ductal carcinoma with lymphoid background

SLN mapping was performed on 10 women as an initial experience. The mean age was 45± 3.6 years. All of these patients were newly diagnosed biopsy-proven cases of early-stage breast cancer planned for breast surgery. Six of these patients had single well-defined outer quadrant lesions, two presented with more than one lesion in a single breast in different quadrants while a single central lesion was identified in the rest of the two patients. Same-day protocol was adopted for each and lymphoscintigraphy was performed three to four hours before breast surgery. SLN localization was successful in all 10 cases with further confirmation on SPECT-CT images. Every time the sentinel node was localized in the ipsilateral axilla. The feedback received from the surgeon was again very encouraging as in all patients gamma probe detected the similar nodes as sentinel as were defined by radionuclide lymphoscintigraphy, which proved to be helpful in surgical decision-making. Sentinel node biopsy in six patients did not reveal metastatic involvement of lymph nodes so the axillary nodal clearance was not done; however, in the remaining four patients, there was histopathological evidence of metastatic nodal involvement as shown in Figure 2).

**Figure 3:**
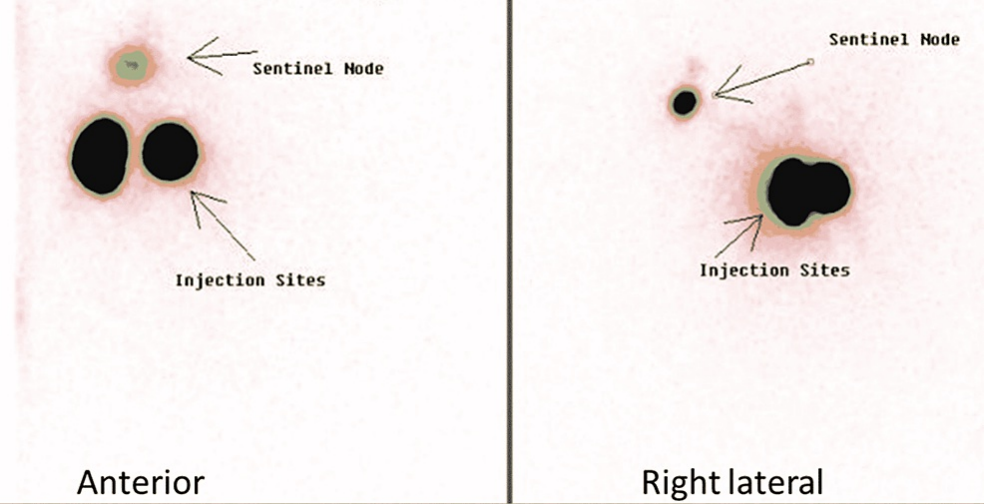
Planar images of SLN mapping with injection sites and sentinel node (anterior and right lateral) SLN: sentinel lymph node

**Figure 4:**
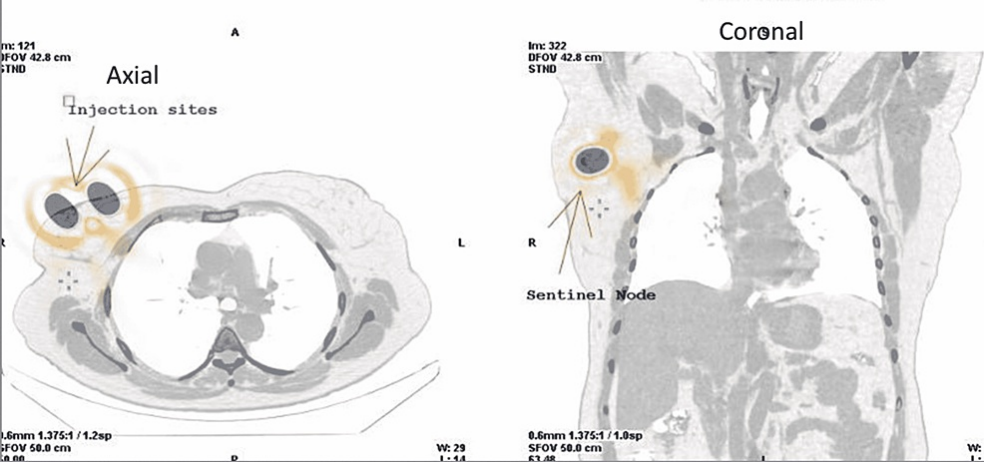
SPECT-CT axial and coronal sections showing injection sites and right axillary sentinel node SPECT: single-photon emission computerized tomography

The planar scintigraphy as shown in Figure 3 demonstrated a prominent focus on radiotracer distribution in the ipsilateral axilla; both peri-areolar injection sites were grossly identified. Higher resolution also revealed tracer transit through lymphatic channels towards the axilla. SPECT-CT images revealed radiotracer avidity in a prominent mildly enlarged rounded lymph node (11 mm) in the ipsilateral axilla as shown in Figure 4. The report concluded successful localization of the ipsilateral axillary lymph node as a sentinel node. In the case of the patient whose images are shown in Figure 2, a maximum number of counts were detected by intraoperative gamma probe from a node that was previously identified as a sentinel on SLN lymphoscintigraphy; hence, it was further confirmed as the sentinel node. The same node and two more in its proximity were removed by the surgeon during the frozen section. As shown in Figure 2, histopathological findings of the frozen section sample revealed metastatic involvement of two lymph nodes (one sentinel and one in its approximation), later on, axillary nodal clearance including excision of an additional 6 to 7 nodes in the vicinity of sentinel node was performed along with mastectomy. This is how radionuclide mapping of sentinel nodes played a significant role in the surgical management of breast cancer patients

## Discussion

Pakistan is considered to have the highest incidence of breast cancer among Asian countries. With increasing awareness, particularly among the urban population of Pakistan, the extent of breast cancer at the time of diagnosis has improved slightly from stage IV to earlier stages, though still statistically insignificant to create an impact on patient management. Recently published reports from the country have documented favorable outcomes of breast conservation surgeries and sentinel node biopsy procedures using an intra-operative probe in early-stage breast cancer [7,8]. The five years of disease-free survival in breast cancer is dependent on the stage of disease at the time of diagnosis, being 85% in early-stage (I and II) breast cancer as compared to 10% in stage IV cases [9]. For the patients diagnosed with early stages of breast cancer, the axillary nodal staging is identified as a critical prognostic factor. The SLN biopsy is recognized as a standard procedure for axillary nodal staging in early breast cancer [9,10]. Accurate detection of the SLN is vital in deciding the treatment plan, which may include or exclude axillary dissection depending on the histological results of the excised sentinel lymph node. The postoperative complication including lymphedema, restricted limb mobility, propensity to develop an infection, and neurological deficit are less in patients undergoing sentinel lymph node biopsy as compared to axillary nodal dissection [11] The main advantage of SLN biopsy is fewer axillary nodes are dissected and thus complications of complete dissection are prevented. Radionuclide lymphoscintigraphy is more frequently being used preoperatively in breast cancer patients as the accuracy and sensitivity of the procedure are better than other techniques [12]. Axillary dissection is thus reserved for patients with positive SLNs on immunohistochemistry or in whom the SLN detection failed [13,14]. There is increased use of SLN procedures in the local settings in the last decade using planar imaging as well as SPECT-CT scanning [15,16].

With the studies indicating a rising incidence of breast cancer in Pakistan [17], a proportionate increase in diagnostic facilities is required; much more than it was in previous years to detect the disease at an early stage and thus reduce the mortality and morbidity associated with the disease and its toxic treatment. The number of nuclear medicine surgical oncology centers offering radionuclide sentinel node detection using gamma probes is very few across the country. In the densely populous capital city of the province of Punjab, there are only two such facilities operating in Lahore. With the addition of such a facility at our center, which is a highly specialized institute offering diagnostic and treatment services for cancer patients, a huge number of patients are expected to benefit. According to the institute registry, an average of 1000 patients are attended to in the breast screening clinic every year and >1200 mammography procedures are performed annually. The center has already established liaisons with five surgical units in the city, which have a high turnover of breast cancer patients. Additionally, training is provided at our center to conduct the procedure successfully, which leads to increased availability of the service in other centers of the country.

The success rate of lymphoscintigraphy and SLN detection was 98.0% in a research study by the International Atomic Energy Agency (IAEA), which concluded that radio-guided SLN biopsy by lymphoscintigraphy and intraoperative gamma probe is feasible and reliable for axillary staging in early breast carcinoma patients [18]. Our study results are quite in line with this, though there are certain limitations.

The purpose of sharing our initial experience with a limited number of patients is to spread the fact that radionuclide SLN mapping is not a very complicated procedure and, to begin with, only a few weeks of training of a closely involved team (doctor and technologists) may be sufficient. Better utilizing already available technical manpower and equipment can be helpful and is the main required component. Although the procedure is relatively simple, many cancer centers in our country have yet not established it. Furthermore, our experience sharing can also be a little help and motivation for other institutes of the country and that of multiple other middle- to low-income countries where the situation is not very different.

Although there are a few examples [19] when research studies with a limited patient population are published, the same factor was considered as a major limitation in this study. However, we are in process of larger data collection and its results will be presented more confidently in the near future, which may help to validate the results of our initial experience. 

## Conclusions

This initial experience of combined procedures for SLN localization and diagnosis of nodal disease infiltration concluded that in multidisciplinary/closely placed surgical, pathological, and hybrid imaging facility settings, lymphoscintigraphy provides a quick, accurate, and better way of nodal localization leading to correct surgical decision making. The planar and SPECT-CT imaging identification of sentinel node followed by nodal biopsy proves to be substantially beneficial for breast cancer patients as it helps to save them from potential adverse effects of surgery.
